# Correlation between Traits of Emotion-Based Impulsivity and Intrinsic Default-Mode Network Activity

**DOI:** 10.1155/2017/9297621

**Published:** 2017-10-31

**Authors:** Jizheng Zhao, Dardo Tomasi, Corinde E. Wiers, Ehsan Shokri-Kojori, Şükrü B. Demiral, Yi Zhang, Nora D. Volkow, Gene-Jack Wang

**Affiliations:** ^1^College of Mechanical and Electronic Engineering, Northwest Agriculture & Forestry University, Yangling, Shaanxi 712100, China; ^2^Laboratory of Neuroimaging, National Institute on Alcoholism and Alcohol Abuse, Bethesda, MD 20892, USA; ^3^Center for Brain Imaging, School of Life Science and Technology, Xidian University, Xi'an, Shaanxi 710071, China; ^4^National Institute on Drug Abuse, Bethesda, MD 20892, USA

## Abstract

Negative urgency (NU) and positive urgency (PU) are implicated in several high-risk behaviors, such as eating disorders, substance use disorders, and nonsuicidal self-injury behavior. The current study aimed to explore the possible link between trait of urgency and brain activity at rest. We assessed the amplitude of low-frequency fluctuations (ALFF) of the resting-state functional magnetic resonance imaging (fMRI) signal in 85 healthy volunteers. Trait urgency measures were related to ALFF in the lateral orbitofrontal cortex, dorsolateral prefrontal cortex, ventral and dorsal medial frontal cortex, anterior cingulate, and posterior cingulate cortex/precuneus. In addition, trait urgency measures showed significant correlations with the functional connectivity of the posterior cingulate cortex/precuneus seed with the thalamus and midbrain region. These findings suggest an association between intrinsic brain activity and impulsive behaviors in healthy humans.

## 1. Introduction

A considerable body of research has documented a strong link between alerted trait urgency and impulsive high-risk behaviors, such as excessive drinking and pathological gambling [1]. For example, negative urgency (NU) has been found to be significantly related to alcohol abuse [2–4], bulimic symptoms [3–5], and nonsuicidal self-injury behavior [2, 4], and positive urgency (PU) has been associated with nonsuicidal self-injury behavior [5], illegal drug use, and risky sexual behavior [6]. Recent longitudinal studies indicate that urgency is a reliable predictor of later risk behavior, for example, the quantity of alcohol consumption during a given drinking episode [7] and the level of alcohol use one year later [8], increases in illegal drug use, and risky sexual behavior [6].

Neuroimaging studies on neuropsychiatric disorders have shown a relationship between the frontal cortex and urgency. Cyders and Smith have documented amygdala, orbitofrontal cortex (OFC, especially the ventromedial prefrontal cortex (vmPFC)) as key neural underpinnings of urgency [9]. Findings from neuroimaging studies in neurological populations also confirm this urgency theory. PU is associated with reduced cortical thickness in the left rostral anterior cingulate cortex (ACC) and right frontal pole, whereas NU shows an inverse association with cortical thickness in the vmPFC and OFC in patients with schizophrenia [10]. In cocaine-dependent individuals, NU correlates positively with gray matter volume (GMV) in the inferior frontal gyrus (IFG) [11]. In contrast, studies in healthy subjects have found that NU showed an inverse association with GMV in the IFG [11], dorsomedial prefrontal cortex (dmPFC), and right temporal pole [12]; regions involved in emotion regulation and decision-making. NU is also found to be correlated with vmPFC BOLD response to alcohol cues in social drinkers, and the BOLD activation in bilateral vmPFC is indirectly associated with increasing subjective alcohol craving and problematic alcohol use during which NU acts as a mediator [13].

However, only a few studies have examined the neurocircuitry underlying trait urgency in healthy individuals [10–12]. Resting-state functional magnetic resonance imaging (rfMRI) [14] has been extensively used to evaluate functional coupling between brain regions during brief (3–6 minutes) MRI scanning [15, 16] and has demonstrated associations between spontaneous brain activity and phenotypic features such as personality traits [17] or mood ratings [18]. Two recent rfMRI studies reported on the association between brain functional connectivity and trait urgency in healthy subjects. In both studies, PU showed a negative correlation with the strength of the functional connectivity within the default-mode network (DMN) [19, 20].

The DMN consists of the medial prefrontal cortex (mPFC), posterior cingulate/precuneus (PCC/precuneus), and angular gyrus, with other midline cortical cortices (such as ACC) and temporal lobe [21, 22]. Prior work has shown that the MPFC and ACC encode salience attribution of rewards and were important for emotion regulation and impulse control [21]. The PCC plays a central role in keeping the balance between internally and externally focused attention [22]. The activity of DMN is modulated by serotonin (5-HT) [23] and dopamine density [24, 25], both of which contribute to emotion-based actions [9]. Taken together, we hypothesized that activity in DMN was related to aspects of urgency trait. The amplitude of low-frequency fluctuations (ALFF) in the brain reflects intrinsic brain low-frequency activity and has been widely used to study cognitive and emotional processes and neuropsychiatric disorders [26]. First, we hypothesized that trait urgency was associated with intrinsic activity measured with ALFF within the DMN. More specifically, trait urgency would be associated with ALFF in medial prefrontal cortices (mPFC) and PCC/precuneus. Second, we hypothesized that NU and PU would correlate with the functional connectivity between the PCC/precuneus, thalamus, and midbrain [22, 27].

## 2. Methods

### 2.1. Participants and Imaging Datasets

The rfMRI datasets of the Nathan Kline Institute [28] were downloaded from the International Neuroimaging Data-sharing Initiative (INDI) online database (http://fcon_1000.projects.nitrc.org/indi/pro/nki.html). All participants provided written informed consent and were scanned according to procedures approved by the local Institutional Review Board (IRB) at the Nathan Kline Institute. The data was shared with the approval of the IRB at the Nathan Kline Institute.

For the current study, 85 healthy subjects (female: 40, male: 45; age range: 18–70, mean ± SD = 38.46 ± 15.55 years) who completed resting state scans and the UPPS-p Impulsive Behavior Scale [29] were included. The UPPS Impulsive Behavior Scale assessed five dimensional characteristics of impulsivity: (1) lack of *premeditation* and (2) *perseverance*, which reflected deficits in conscientiousness to act without forethought or to tolerate a boredom task; (3) *sensation-seeking*, which reflected the reward-sensitive; and (4) *negative urgency* and (5) *positive urgency*, which reflected the emotion-based disposition to engage in rash actions [29]. Subjects with a history of psychiatric disorders or medical conditions were excluded. In addition, subjects with Beck Depression Inventory (BDI) [30] scores higher than 15, indicating mild-severe depression, were excluded.

Structural MRI scans were acquired with a Siemens MAGNETOM Tim Trio 3.0 T Scanner (TR = 2.5 s; TE = 3.5 ms; TI = 1200 ms; FOV: 256 × 256; slice thickness: 1 mm; flip angle: 8°; matrix size: 256 × 256; 200 transverse slices). Functional MRI images were acquired with a gradient echo-planar sequence (TR = 2.5 s; TE = 35 ms; flip angle = 80°; FOV: 256 × 256; in-plane resolution = 3 × 3 mm^2^, slice thickness: 3 mm, 260 time points = 10.83 min).

### 2.2. fMRI Preprocessing

Functional data were analyzed using SPM8 (Welcome Department of Cognitive Neurology, London, UK, http://www.fil.ion.ucl.ac.uk/spm). Image preprocessing included slice-time correction, image realignment, and spatial normalization to the stereotactic space of the Montreal Neurological Institute (MNI) and resampling to 3 mm isotropic voxels. Head motion correction was conducted based on a “scrubbing” approach [31]. Specifically, if the framewise displacement (FD) was larger than 0.5 mm or the root mean square signal change (DVARS) was larger than 5%, the corresponding volume was linearly interpolated using its temporal neighbors [31]. Multiple linear regression was performed to remove nuisances such as the mean signal fluctuations in the whole brain, ventricles and white matter, and the six head realignment parameters and their derivatives. Detrending and a temporal band-pass filtering (0.01–0.08 Hz) were subsequently conducted to minimize temporal drifts and white noise.

### 2.3. ALFF

ALFF maps were calculated for each subject by using DPARSF toolbox [32]. ALFF was defined as the total power within the frequency range between 0.01 and 0.08 Hz, which represented the strength or intensity of low-frequency oscillations [32, 33].

### 2.4. Seed-Voxel Correlations

**Figure 1:**
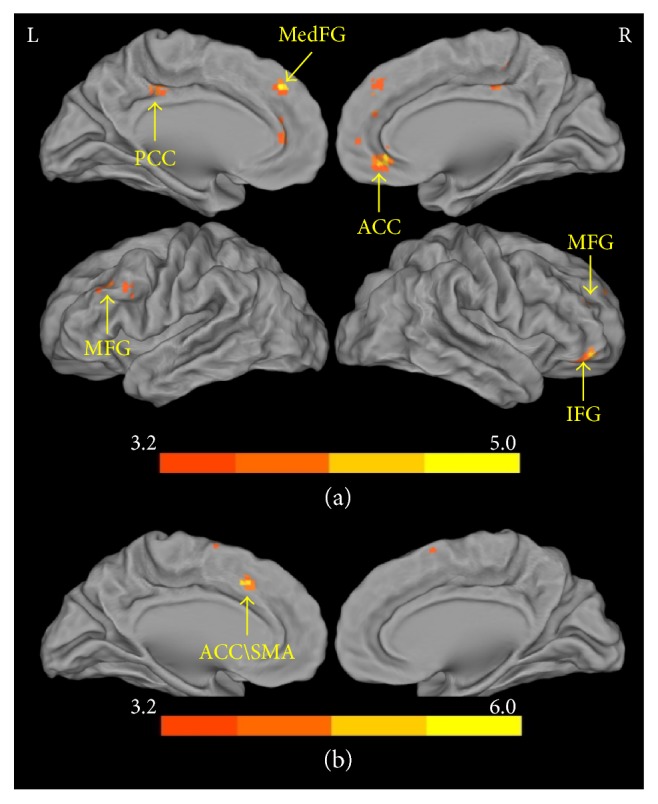
Brain mapping of intrinsic activity demonstrates a significant association with trait urgency (*P*_FWE_ = 0.05, family-wise error correction). (a) The brain regions indicate correlations with mean scores of negative and positive urgency. (b) The brain regions indicate stronger correlations with negative urgency than with positive urgency. PCC: posterior cingulate cortex; MedFG: medial frontal gyrus; ACC: anterior cingulate cortex; MFG: middle frontal gyrus; IFG: inferior frontal gyrus; SMA: supplementary motor area.

The PCC/precuneus [xyz = (−6, −28, 34) mm; cluster volume = 52 voxels] and subgenual ACC [xyz = (−6, 41, −2) mm; cluster volume = 107 voxels] were selected for seed-voxel correlation analysis (see Figure 1). In order to reduce the risk of circularity analysis, two independent masks were employed to be seed regions to conduct the functional connectivity analyses. The first brain mask for the seed was derived from the Human Brainnetome Atlas [34] (bilateral CG-6 region, supplementary figure 1a available online at https://doi.org/10.1155/2017/9297621). The second brain mask for the seed was anatomically defined by bilateral dorsal posterior cingulate cortex [35] (supplementary figure 1b). The DPARSF toolbox was used to calculate seed-voxel correlations [32], and the Pearson correlation maps were converted to Fisher z-scores prior to statistical analyses.

### 2.5. Statistical Analyses

Statistical analyses were performed using SPM8 (Welcome Department of Cognitive Neurology, London, UK; http://www.fil.ion.ucl.ac.uk/spm). Multiple linear regression was used to assess the association between urgency and functional connectivity metrics (ALFF and seed-voxel correlation maps). Given the high correlation between scores of NU and PU (*r* = 0.702, *P* < 0.001), the mean scores of NU and PU [(NU + PU)/2] and the difference between scores of NU and the mean scores [(NU − PU)/2] were entered in each statistical model. In addition, the subject's age and gender were included as covariates. Statistical significance was based on a family-wise error (FWE) correction for multiple comparisons at the cluster-level (*P*_FWE_ < 0.05) with a minimum cluster size of *k* = 30 voxels and a cluster-defining threshold *P* < 0.001 [36, 37].

## 3. Results

### 3.1. ALFF

Table 1 shows that the mean scores of NU and PU were positively correlated with ALFF in the subgenual ACC (Brodmann areas (BA) BAs 32 and 11, sgACC), dorsal ACC (dACC, BA 32), medial frontal gyrus (BAs 10 and 8, mPFC), right middle frontal gyrus (BA 46, DLPFC), right IFG (BA 47), PCC/precuneus (BAs 23 and 31), left IFG (BA 44), and MFG (BAs, 8 and 9). There was no region in which intrinsic activity showed negative correlation with mean scores of NU and PU. Intrinsic activity in ACC (BA 32) and supplementary motor area (BA 6) showed tighter positive correlation with NU than PU. There was no region which intrinsic activity showed tighter positive correlation with PU than NU (Figure 1, Table 1).

### 3.2. Functional Connectivity

**Figure 2:**
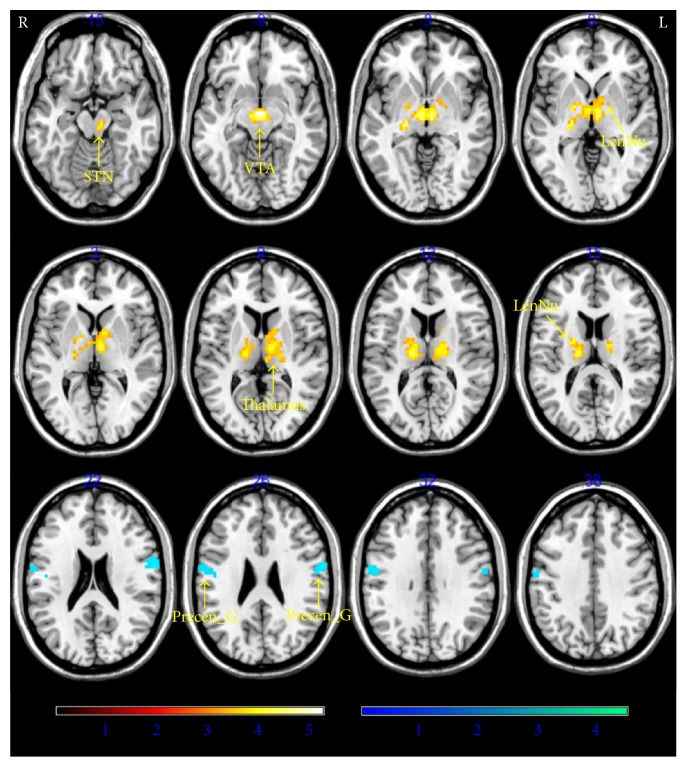
Brain mapping of functional connectivity with PCC/precuneus demonstrates a significant association with mean scores of negative and positive urgency (*P*_FWE_ = 0.05, family-wise error correction). The hot color indicates a positive correlation with mean scores of negative and positive urgency; the cool color indicates a negative correlation with mean scores of negative and positive urgency. STN: substantia nigra; VTA: ventral tegmental areas; LenNu: lentiform nucleus; Precen_G: precentral gyrus.

The functional connectivity of the PCC/precuneus seed with the rest of the brain showed a significant positive correlation for mean scores of NU and PU with ventral tegmental areas of the midbrain, thalamus, lentiform nucleus of lateral globus pallidus, and medial globus pallidus, putamen, substantia nigra, and caudate (Table 2, Figure 2). The functional connectivity of the PCC/precuneus seed with the rest of the brain showed a significant negative correlation for mean scores of NU and PU with bilateral precentral gyrus (BAs 4 and 6). With regard to the functional connectivity with the PCC/precuneus, there was no region showing a significant different association between NU and PU (Figure 2, Table 2). No brain region showed that its functional connectivity with sgACC seed was significantly associated with urgency trait.

Both of those two functional connectivity analyses with independent seeding mask replicated the former findings that the trait urgency was positively associated with functional connectivity between PCC and thalamus midbrain regions (supplementary figure 1).

## 4. Discussion

Here, we report an association between trait urgency and ALFF within the DMN. Specifically, we found that ALFF in sgACC, mPFC, right DLPFC, left IFG and MFG, and PCC/precuneus were positively correlated with urgency. Furthermore, NU and PU showed significant correlations with the functional connectivity between PCC/precuneus and thalamus, as well as the functional connectivity between PCC/precuneus and substantia nigra and ventral tegmental areas of the midbrain, which is where dopamine neurons are located.

From a theoretical perspective on urgency, Cyders and Smith propose that the amygdala and vmPFC are involved in emotion-based rash action [9]. The amygdala encodes emotional significance of sensory input and influences subsequent cortical activity for cognitive processing. Meanwhile, amygdala affects the activity of the nucleus accumbens, and the ventral tegmental area to influence emotional experience and actions [38]. The vmPFC takes charge of regulating effects on the amygdala to provide information toward long-term goal-directed behavior [38]. Consistent with this theoretical point of view [9], we found that the intrinsic activity of sgACC and mPFC was associated with trait urgency (Figure 1). In addition, we found that trait urgency was associated with the amplitude of functional connectivity between PCC/precuneus and thalamus, responding for sensory processing, as well as between PCC/precuneus and midbrain regions for reward-related cognitive processing. These findings indicate that trait urgency is related to the dopamine reward system.

These results are consistent with previous findings showing an association between trait urgency and brain activation in the right lateral OFC during the presentation of negative emotional stimuli [39]. The lateral OFC is involved in motivation and reward sensitivity and has been suggested to inhibit neural processing associated with irrelevant sensations and actions when emotion influences attention [40]. A neuroimaging study in children with attention-deficit/hyperactivity disorder (ADHD) has found enhanced local connectivity in the lateral OFC for ADHD children compared to controls, which was interpreted to reflect lower dopaminergic function [41]. Furthermore, increased OFC activation was consistently found during negative and positive emotional processing [42, 43]. These results hence suggest that urgency is related to the regulatory activity of the lateral OFC. Although Cyders et al. find no association between the lateral OFC's response to positive images and PU [39], our findings indicate a link between urgency and resting OFC activity.

Our findings showed that both PU and NU were associated with the intrinsic activity in PCC/precuneus. The PCC/precuneus is a hub region of the DMN involved with high order information processing [44, 45]. A large body of literature has documented that activity in PCC/precuneus is suppressed during cognitive processes, such as attention [46, 47], memory [47], and decision-making [48, 49]. The magnitude of activity changes in PCC/precuneus has been related to task difficulty [50], and inappropriate deactivation in PCC/precuneus has been associated with weaker performances during cognitive tasks [51–53]. Previous studies have shown a strong association between dopaminergic function and brain activity in the PCC/precuneus [41]. Specifically, activation of PCC/precuneus during a visual attention task is shown to increase with increased dopamine transporter levels in healthy volunteers [54]. Moreover, L-dopa administration is documented to decrease functional connectivity between PCC/precuneus and caudate, as compared to baseline [55]. Previous studies also show a positive correlation between resting state activity in the precuneus and personality traits of sensitivity to external environmental and social conditions [56]. Since urgency reflects poor cognitive control when facing salient stimuli, enhanced resting PCC activity in healthy participants may also indicate an alteration of reward-related cognitive function, which may contribute to the emergence of urgency behavior.

We further showed that trait urgency was associated with intrinsic activity in DLPFC and dACC. DLPFC is connected with premotor areas, lateral parietal cortex, and thalamus [57, 58], which are areas associated with cognitive control and executive function [59]. DLPFC takes part in rule-based working memory and top-down cognitive control, and dACC is involved in conflict monitoring [60]. Both areas are activated in emotion reappraisal tasks, indicative of their role in cognitive control [42], as well as in emotion regulation [61]. The positive relationship between the intrinsic activity of the DLPFC and the dACC with trait urgency may indicate that fast-acting emotional processes accompany an altered baseline activation of the cognitive control system. Given the functional connectivity between PCC and ACC and the structural connections between thalamus and DLPFC, future studies may use path analyses to further explore the augmented baseline activity in the cognitive control system for individuals with high trait PU.

In the current study, trait urgency was related to the functional connectivity between PCC and the thalamus, as well as the functional connectivity between PCC and substantia nigra and ventral tegmental areas of the midbrain. The thalamus is a relay for striatocortical and corticocortical communication [62] and a number of studies have implicated the thalamus in trait impulsivity. That is, increased volume of the anterior thalamus is associated with higher impulsivity in 14~15-year-old children [63], and impulsivity correlated negatively with functional connectivity between the thalamus and the ventral striatum [64]. In our study, both NU and PU show correlation with the functional connectivity between PCC and the medial dorsal, lateral posterior, and ventral lateral nuclei of the thalamus. The medial dorsal nucleus of the thalamus receives inputs from the amygdala and the olfactory cortex [27]. The lateral posterior and ventral lateral nuclei of the thalamus receive input from substantia nigra and globus pallidus and project to the premotor cortex [27]. In turn, the precuneus receives connections from the occipital, temporal, and posterior parietal subdivisions of the thalamus for sensory information translation [65] and their disruption might contribute to rash action. The amplitude of structural connectivity between PCC/precuneus and thalamus has also been associated with impairment of consciousness [66]. A recent study finds linear associations between dopamine D2/D3 receptors in the striatum and fMRI signals in the thalamus and precuneus during a visual attention task, indicative of the role of dopaminergic rewarding effects on the activity of thalamus and precuneus on cognitive functioning [54]. These studies provide evidence of a key role of PCC/precuneus and thalamus in consciousness and cognitive behaviors. Taken together with the results of the current study, the enhanced functional synchronization between PCC/precuneus and thalamus for high trait impulsivity individuals is in line with the proposed role for this circuitry in processes of reward-related information processing and further highlights the contributions of these two regions in the formation of impulsive responses when facing external emotions.

We found that urgency was associated with extensive areas in the medial prefrontal cortex (sgACC, mPFC), which was necessary for autobiographical and self-referential processing [67]. The mPFC is anatomically and functionally connected with the amygdala, which is involved in the detection and evaluation of emotional salience [68].

The association between NU and mPFC (ACC/supplementary motor area) is stronger than that between PU and mPFC, which may be related to the key role of mPFC in negative emotion processing [69]. These results implicate different brain networks underlying NU and PU, and further studies aim at replicating this finding may help better understand the overlapping and distinct neurocircuitry of PU and NU.

In the current study, we found that trait urgency was associated with the intrinsic brain activity in sgACC and vmPFC in response to emotion experience [70]. Notably, traits related to urgency are also related to the intrinsic brain activity of lateral OFC, DLPFC, and dACC, which are recruited in successful emotional regulation [21]. The concept of urgency describes a process which links emotion and many different risky behaviors. In line with the theory of urgency, the association of trait urgency and brain activity in those regions confirms that the dysfunction of emotion regulation may contribute to the rash action under intense emotion condition, which may suggest that it is possible to employ emotion regulation strategy to ameliorate trait urgency to avoid risky behaviors [71].

## 5. Limitation

In the current study, there are some limitations that should be noted. First, we employed a cross-sectional design to explore the possible link between trait urgency and brain intrinsic activity. The cross-sectional design made it difficult to clarify any causal relationships. For example, we could not clarify whether the increase of intrinsic activity of sgACC/vmPFC will enhance emotional-based rash action. Further longitudinal studies with neuroimaging technology should be conducted to identify the causal effect of brain activity and trait urgency. Second, we did not perform a test-retest examination on an independent data due to unavailability of a similar resting fMRI data with UPPS scale. Future studies are needed to replicate our findings.

## 6. Conclusion

The current study employs ALFF and functional connectivity methods to explore the signatures of NU and PU in intrinsic activity of the brain. NU and PU are related to activity of frontal and cingulate cortices and are associated with the functional connectivity between the PCC/precuneus and thalamus and with the functional connectivity between the PCC/precuneus and midbrain. In summary, we provide evidence that aspects of intrinsic brain activity are related to urgency in behavior and may shed light on the neural mechanism underlying impulsivity.

## Supplementary Material

Supplementary Figure 1 the brain regions which functional connectivity with dorsal posterior cingulate cortex showed significant association with trait urgency (P_FWE_ = 0.05=, family-wise error (FWE) correction). (a) Seed region was defined from the Human Brainnetome Atlas (bilateral CG-6 region, L. Fan, 2016). (b) Seed region was anatomically defined by bilateral dorsal posterior cingulate cortex (B. A. Vogt, 2006).

## Figures and Tables

**Table 1:** The foci of brain areas showed intrinsic activity associating with trait urgency (*P*_FWE_ = 0.05, family-wise error correction).

Region	Brodmann area	Voxel	*Z*	MNI		
				*X*	*Y*	*Z*
*Brain regions in which intrinsic activity showed positive correlation with mean score of negative urgency and positive urgency*						
Anterior cingulate	BA 11	107	4.55	6	41	−2
Anterior cingulate	BA 32		4.04	−12	35	16
Medial frontal gyrus	BA 10		3.84	12	50	4
Middle frontal gyrus	BA 46	45	4.35	27	35	16
Cingulate gyrus	BA 32		3.31	24	29	28
Medial frontal gyrus	BA 8	63	4.29	−6	38	37
Superior frontal gyrus	BA 8		4.15	9	38	40
Medial frontal gyrus	BA 8		3.98	15	50	31
Inferior frontal gyrus	BA 47	42	4.22	39	47	−5
Inferior frontal gyrus	BA 47		3.73	45	35	−11
Cingulate gyrus	BA 23	52	4.2	−6	−28	34
Cingulate gyrus	BA 31		3.98	3	−25	40
Cingulate gyrus	BA 31		3.78	9	−31	37
Middle frontal gyrus	BA 8	54	4.17	−30	20	31
Inferior frontal gyrus	BA 44		4.12	−33	11	34
Middle frontal gyrus	BA 9		3.65	−36	26	34
*Brain regions in which intrinsic activity showed negative correlation with mean score of negative urgency and positive urgency*						
No region survived						
*Brain regions in which intrinsic activity showed tighter correlation with negative urgency than positive urgency*						
Anterior cingulate	BA 32	60	5.12	−6	20	40
Supplementary motor area	BA 6		4.58	0	11	52
Supplementary motor area	BA 6		3.51	−3	5	58
*Brain regions which intrinsic activity showed tighter correlation with positive urgency than negative urgency*						
No region survived						

Note: BA = Brodmann areas.

**Table 2:** The foci of brain areas in which functional connectivity with PCC/precuneus was significantly correlated with negative or positive urgency (*P*_FWE_ = 0.05, family-wise error correction).

Region	Brodmann area	Voxel	*Z*	MNI		
				*X*	*Y*	*Z*
*Brain regions which functional connectivity with PCC/precuneus are positively associated with negative and positive urgency*						
Ventral tegmental areas	Mammillary body	487	4.82	0	−9	−9
Thalamus	^∗^		4.51	6	−12	−3
Thalamus	^∗^		4.44	−21	−21	−3
Thalamus	^∗^		4.34	−6	−12	−3
Thalamus	Ventral lateral nucleus		4.14	−15	−15	12
Lentiform nucleus	Lateral globus pallidus		3.96	−18	−3	0
Thalamus	Medial dorsal nucleus		3.93	9	−12	9
Lentiform nucleus	Putamen		3.91	−21	−6	15
Thalamus	^∗^		3.74	12	3	3
Thalamus	Ventral lateral nucleus		3.72	18	−15	12
Caudate	Caudate body		3.68	−21	−18	24
Substantia nigra	^∗^		3.68	9	−21	−15
Thalamus	Lateral posterior nucleus		3.57	21	−18	9
Thalamus	^∗^		3.45	6	−24	9
Caudate	Caudate body		3.4	15	6	9
Lentiform nucleus	Medial globus pallidus		3.27	18	0	−3
*Brain regions which functional connectivity with PCC/precuneus are negatively associated with negative and positive urgency*						
Precentral gyrus	BA 4	69	4.21	63	−3	24
Precentral gyrus	BA 4	75	3.73	−57	−6	33
Precentral gyrus	BA 4		3.63	−60	−6	24
Precentral gyrus	BA 6		3.37	−60	−9	42

Note: ^∗^No BA covered. BA = Brodmann areas.
