# Supplementary material for: Correlation between Traits of Emotion-Based Impulsivity and Intrinsic Default-Mode Network Activity
Source: Neural Plast. 2017 Oct 31;2017:9297621. doi: 10.1155/2017/9297621 (PMC5684566; doi:10.1155/2017/9297621)
Supplement: Supplementary file 1 — Supplementary Figure 1 the brain regions which functional connectivity with dorsal posterior cingulate cortex showed significant association with trait urgency (PFWE = 0.05=, family-wise error (FWE) correction). (a) Seed region was defined from the Human Brainnetome Atlas (bilateral CG-6 region, L. Fan, 2016). (b) Seed region was anatomically defined by bilateral dorsal posterior cingulate cortex (B. A. Vogt, 2006). [file 9297621.f1.pdf]

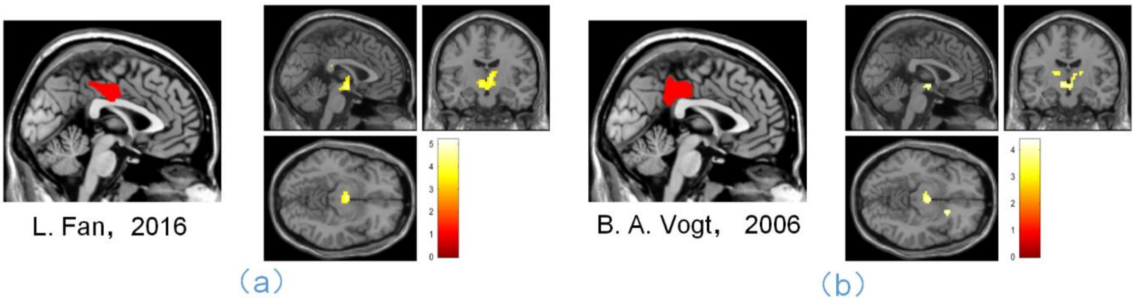

Supplementary Figure 1 the brain regions which functional connectivity with dorsal posterior cingulate cortex showed significant association with trait urgency (P_{FWE} = 0.05, family-wise error (FWE) correction). (a) Seed region was defined from the Human Brainnetome Atlas (bilateral CG-6 region, L. Fan，2016). (b) Seed region was anatomically defined by bilateral dorsal posterior cingulate cortex (B. A. Vogt，2006).
